# Emergency laparoscopic cholecystectomy for intraabdominal hemorrhage in a patient with a left ventricular assist device: a case report

**DOI:** 10.1186/s40792-019-0756-9

**Published:** 2019-12-11

**Authors:** Akihiko Takagi, Erina Nagai, Takeo Toda, Hayato Kosaka, Hisato Ishimatsu, Yusuke Kyoden, Takehide Akimoto, Hideyuki Kanemoto, Noriyuki Oba

**Affiliations:** 1 Department of Gastroenterological Surgery, Shizuoka General Hospital, 4-27-1 Kita-ando, Aoi-ku, Shizuoka-shi, Shizuoka, 420-8527 Japan; 2 Department of Cardiovascular Surgery, Shizuoka General Hospital, Shizuoka, Japan

**Keywords:** Intraabdominal hemorrhage, Laparoscopic cholecystectomy, Ventricular assist device

## Abstract

**Background:**

Continuous-flow left ventricular assist devices (LVADs), called “second generation LVADs,” have significantly improved the survival and quality of life outcomes. Accordingly, non-cardiac surgery in a patient with LVADs has required for conditions not directly related to their LVADs. And the management of bleeding in non-cardiac site remains one of long-term critical topics. Laparoscopic approach is useful in a patient with LVADs; however, there have been only few clinical reports. This report describes the first case of laparoscopic cholecystectomy (LC) for intraabdominal hemorrhage from the gallbladder serosa in a patient with LVADs.

**Case presentation:**

A 56-year-old man with an LVAD had undergone LVAD (Jarvik 2000™; Jarvik Heart, Inc., New York, NY, USA) implantation at 53 years of age. He was in shock, and contrast-enhanced computed tomography revealed abdominal hemorrhage from the gallbladder serosa. Emergency laparoscopic cholecystectomy was performed. We could avoid injury of the LVADs driveline, which was located across the upper abdominal midline, near the right hypochondriac region, by laparoscopic approach. LVADs (Jarvik 2000) did not disturb the operating field because of its smaller size. There were no intra- and postoperative complications.

**Conclusions:**

Laparoscopic approach is useful and safe in a patient with LVADs for abdominal surgery. We could perform LC for intraabdominal hemorrhage from gallbladder serosa safety.

## Background

**Fig. 1:**
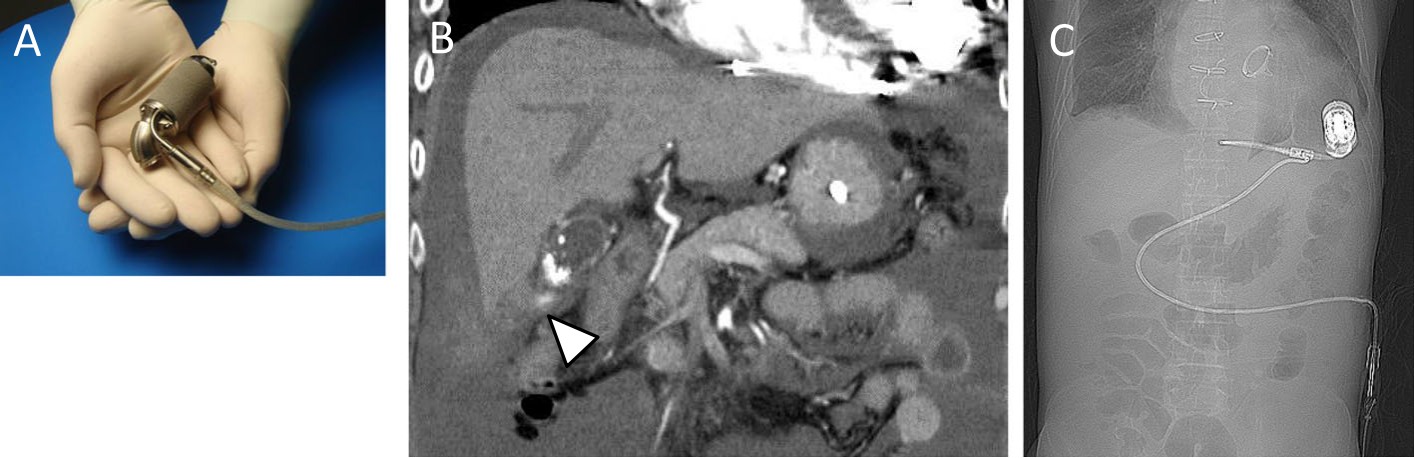
Diagnostic images and a photograph of LVAD. **a** JarvikTM 2000. **b** Abdominal radiographs taken on admission showing the LVAD main body and driveline location. **c** Coronal computed tomography scan showing a high-density area near the gallbladder (arrowhead), high-density ascites, and gallbladder stone. There is no high-density area in the gallbladder

Continuous-flow left ventricular assist devices (LVADs), called “second generation LVADs,” have significantly improved the survival and quality of life outcomes [1]. However, they have been associated with the development of non-cardiac complications over time [2]. In abdominal surgery, it is very important to avoid injury of the LVAD driveline, which connects the main body with the controller running under the abdominal skin. Thus, laparoscopic approach is useful in these patients; however, there have been only few clinical reports. We herein report a first case of emergency laparoscopic cholecystectomy (LC) for intraabdominal hemorrhage from the gallbladder serosa in a patient with LVADs (Jarvik 2000™; Jarvik Heart, Inc., New York, NY, USA). Jarvik 2000 is a second-generation LVAD that features a miniaturized intraventricular pump (Fig. 1a).

## Case presentation

**Fig. 2:**
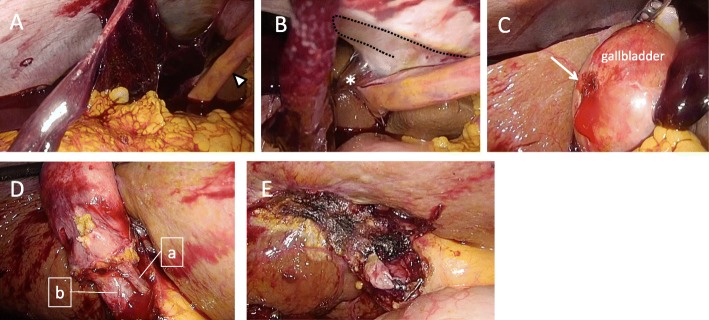
Intraoperative laparoscopic views. **A** Wide-spread hemorrhagic ascites in the sub-hepatic area. The arrowhead indicates the round ligament. The presence of the LVAD main body and driveline did not obstruct the operating field. **B** Inserting the epigastric port (asterisk) while avoiding injury to the driveline (dotted line). **C** The bleeding point (arrow) was at the gallbladder serosa. **D** Standard view of LC: cystic artery (a) and cystic duct (b). **E** View after gallbladder removal

**Fig. 3:**
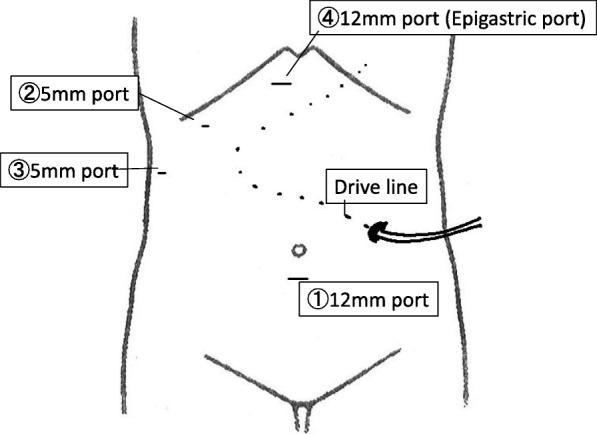
Schema of the ports and driveline. We inserted the ports in order from ① to ④

**Fig. 4:**
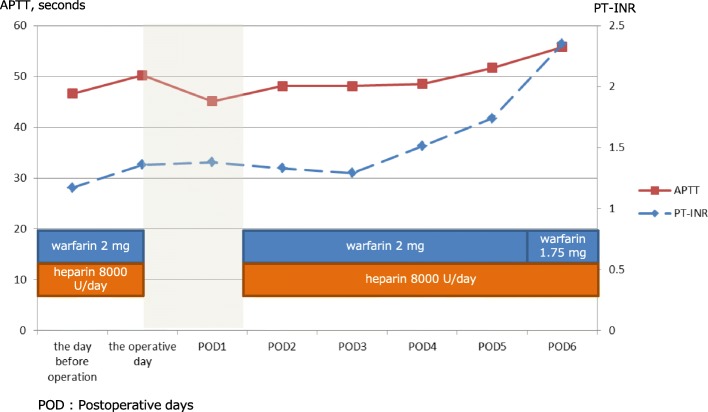
Perioperative anticoagulation management

A 56-year-old man was admitted to our hospital due to right heart failure and epistaxis that could not be controlled by pressure hemostasis. He had undergone LVAD (Jarvik 2000) implantation at 53 years of age due to severe heart failure after cardiovascular surgery (mitral valve replacement due to mitral regurgitation after mitral valve plasty and tricuspid annuloplasty for dilated cardiomyopathy). At the time of admission, he was on anticoagulation therapy with only oral warfarin that had maintained the prothrombin time-international normalized ratio (PT-INR) between 2.5 and 3.5. He had undergone mechanical ventilation therapy for heart failure and had a nasogastric tube inserted. Warfarin was administered through the tube. On day 4 after admission, the patient developed epistaxis. External carotid artery ligature and transcatheter coil embolization were performed for hemostasis. At that time, warfarin was discontinued and neutralized by menatetrenone (vitamin K). After the hemostatic treatments, warfarin was re-started with heparinization. The patient was weaned off mechanical ventilation on day 11 after admission. On day 15 after admission, he developed hypotension, abdominal distension, and mild abdominal pain. Blood tests revealed aggravating anemia; the serum hemoglobin dropped from 10.5 to 8.3 g/dL in 1 day. Contrast-enhanced computed tomography revealed abdominal hemorrhage. Signs of extravasation at the gallbladder wall and a gallstone were seen, but there were no signs of intra-gallbladder hemorrhage or acute cholecystitis (Fig. 1b). Abdominal X-rays were performed to confirm the LVAD driveline location; it was located across the upper abdominal midline, near the right hypochondriac region (Fig. 1c). At that time, the PT-INR and activated partial thromboplastin time were 1.36 and 50.2 s, respectively. We planned to perform emergency laparoscopic cholecystectomy; however, we had to consider the possibility of conversion to open surgery in case of insufficient operating space due to the hemorrhage and LVAD. Therefore, we prepared for laparotomy using X-ray fluoroscopy. We did not use menatetrenone or protamine sulfate for neutralization preoperatively. Before the start of the surgery, we made markings on the skin along the driveline by palpation to avoid its injury at trocar insertion. After the induction of general anesthesia with an arterial pressure line and a central venous catheter, the first trocar for the endoscope was inserted using the open approach (20-mm horizontal incision at the lower edge of the umbilicus). Pneumoperitoneum was created at 10 mmHg, and a 0° endoscope was inserted. On intraabdominal observation, abundant hemorrhagic ascites (a total of about 3000 ml) was seen in the abdominal cavity (Fig. 2A). The other ports were inserted as presented in Fig. 3, with the epigastric port inserted carefully while watching from the intraperitoneal side (Fig. 2B). As noted on the preoperative images, the bleeding point was at the gallbladder wall serosa (Fig. 2C). We were able to stop the bleeding by coagulation. The gallbladder wall had mild inflammatory changes, but there were no adhesions around the gallbladder. There was no finding of a perforation or bile leakage. Although the tissue was fragile and friable, we could perform standard LC (Fig. 2D). During the surgery, the main body and the driveline of the LVAD did not obstruct the operating field. The cystic duct and artery were clipped with a 10-mm clip applicator (Fig. 2E). After exploring the whole abdominal cavity, we confirmed no other bleeding points and no injury to other organs. We placed a drain in the subhepatic space and ended the operation without intraoperative complications (operation time, 1 h 59 min; intraoperative bleeding, 240 ml). The postoperative course was uneventful, and the drain was removed on postoperative day 2. On the same day, warfarin and heparin administration was re-started. The perioperative anticoagulation management and the trend of PT-INR and APTT (seconds) are presented in Fig. 4. After heart failure treatment, the patient was discharged home on postoperative day 30. No changes were made to the anti-coagulation management. At discharge, the dose of warfarin was 1.5 mg/day and PT-INR was 2.59. The pathological findings were gallbladder hemorrhage and chronic cholecystitis with a stone. The main area of hemorrhage was in the subserous tissue.

## Discussion

This case further supports the usefulness of laparoscopic approach for avoiding driveline injury in patients with LVADs. There are currently three case reports [3–5] and three case series including four to six cases [6–8] (Table 1) on laparoscopic cholecystectomy (LC) in patients with an LVAD. There were no cases of conversion to open surgery or critical intraoperative complications. Postoperative complications were reported in two patients who had acute kidney dysfunction and abdominal wall hematoma. All cases were reported on patients with a second- or third-generation LVAD. There are only two case reports of emergency LC, including our case. Naito et al. reported on emergency LC in a patient with an LVAD [5], but the type was DuraHeartTM (Termo Heart, Inc., AnnArbor, MI, USA). Compared with our case, a protruding main body and driveline of the LVAD and mild to severe adhesions to them were observed. We thus speculate that, almost certainly, laparoscopic surgery is easier to perform in patients with Jarvik 2000 than in those with other types of LVADs because of its smaller size.

**Table 1:** Reports of laparoscopic cholecystectomy in patients with a left ventricular assist device

Author(s)	Year	Number of cases	Diagnosis (number of cases)	LVAD type	Conversion	Postoperative complications
Kartha et al. [3]	2008	1	Cholelithiasis	HeartMate II^†^	No	No
Amir et al. [4]	2012	1	Cholecystitis and pancreatitis	HeartMate II^†^	No	No
Naito et al. [5]	2013	1	Cholecystitis	DuraHeart^‡^	No	No
Ashfaq et al. [6]	2016	4	Cholecystitis (3), gallstone pancreatitis with symptomatic cholelithiasis (1)	HeartMate II^†^	No	1 (acute kidney injury)
Suresh et al. [7]	2019	5	Cholelithiasis (4), cholecystitis (1)	NA	No	1 (abdominal wall hematoma)
Vigneswaran et al. [8]	2019	6	Cholelithiasis (4), cholecystitis (1), gallstone pancreatitis (1)	HeartMate II^†^ and HeartWare^§^	No	No
Our case	2019	1	Abdominal hemorrhage	Jarvik 2000	No	No

*NA* not applicable, *LVAD* left ventricular assist device

^†^HeartMate II™ (Thoratec Corp., Pleasanton, CA); second-generation LVAD

^‡^DuraHeart™ (Termo Heart, Inc., AnnArbor, MI); third-generation LVAD

^§^HeartWare™ (International Inc., Framingham, MA); third-generation LVAD

Furthermore, the diagnosis in our case was hemorrhage and the bleeding point was the gallbladder serosa without perforation, severe cholecystitis. There was no traumatic episode; he had been in bed for treatment of heart failure and uncontrollable epistaxis. This finding is extremely rare, and there have been no similar reports. The factor of chronic cholecystitis by gallstones is one of the reasons, but only that usually do not occur intraabdominal hemorrhage. It remains possible that factors peculiar to a patient with LVAD contribute to the condition. The reasons for increased risk of bleeding, particularly gastrointestinal bleeding (GIB), in these patients are reported to be acquired Von Willebrand syndrome and arteriovenous malformation (AVM) [9]. The continuous-flow ventricular pump of these LVADs induces shear stress to the blood vessels, which does not exist in a physiological heart movement, and reduces Von Willebrand factor activity by loss of high-molecular-weight multimers. And approximately 33–50% of all GIB episodes in patients with LVADs are reported to be due to AVMs. In these cases, angiogenesis-related signaling cascade leading to angiodysplasia caused by tumor necrosis factor alpha has been reported as the underlying mechanism. Interestingly, the patient also had refractory epistaxis, which has been reported to be caused by AVMs in patients with second-generation LVADs [9]. However, neither the preoperative computed tomography nor the pathological findings revealed an AVM; this may be due to the intraoperative coagulation.

## Conclusion

Laparoscopic approach is useful and safe in a patient with LVADs for abdominal surgery. We could perform LC for intraabdominal hemorrhage from gallbladder serosa safety. Jarvik 2000, type of LVAD, did not disturb the operating field because of its smaller size.

## Data Availability

There is no available data and materials to be shared.

## Abbreviations

AVM: Arteriovenous malformation
GIB: Gastrointestinal bleeding
LC: Laparoscopic cholecystectomy
LVAD: Left ventricular assist device
PT-INR: Prothrombin time-international normalized ratio

## References

[1] Slaughter MS, Rogers JG, Milano CA (2009). Advanced heart failure treated with continuous-flow left ventricular assist device. N Engl J Med.

[2] Stehlik J, Nelson DM, Kfoury AG (2009). Outcome of noncardiac surgery in patients with ventricular assist devices. Am J Cardiol.

[3] Kartha V, Gomez W, Wu B, Tremper K (2008). Laparoscopic cholecystectomy in a patient with an implantable left ventricular assist device. Br J Anaesth.

[4] Amir O, Bitterman A, Eden A (2012). Laparoscopic cholecystectomy in a left ventricular assist device-supported patient. Isr Med Assoc J.

[5] Naitoh T, Morikawa T, Sakata N, Unno M, Akiyama M, Saiki Y (2013). Emergency laparoscopic cholecystectomy for a patient with an implantable left ventricular assist device: report of a case. Surg Today.

[6] Ashfaq A, Chapital AB, Johnson DJ, Staley LL, Arabia FA, Harold KL (2016). Safety and feasibility of laparoscopic abdominal surgery in patients with mechanical circulatory assist devices. Surg Innov.

[7] Suresh V, Bishawi M, Bryner B (2019). Outcomes of laparoscopic cholecystectomy in patients supported with a left ventricular assist device. J Laparoendosc Adv Surg Tech A.

[8] Vigneswaran Y, Wang V, Krezalek M (2019). Laparoscopic procedures in patients with cardiac ventricular assist devices. Surg Endosc.

[9] Imamura T, Kinugawa K, Uriel N (2018). Therapeutic strategy for gastrointestinal bleeding in patients with left ventricular assist device. Circ J.

